# Management of Head and Neck Pseudoaneurysms: A Review of 33 Consecutive Cases

**DOI:** 10.1155/2014/419803

**Published:** 2014-10-27

**Authors:** Eliza Anderson, Nohra Chalouhi, Aaron Dumont, Stavropoula Tjoumakaris, Mario Zanaty, Robert Rosenwasser, Robert M. Starke, Pascal Jabbour

**Affiliations:** ^1^Department of Neurological Surgery, Thomas Jefferson University, Philadelphia, PA, USA; ^2^Department of Neurological Surgery, University of Virginia, Charlottesville, VA, USA

## Abstract

*Background*. Endosaccular coiling, vessel occlusion, stenting, stent-assisted coiling, and flow diversion are all endovascular treatment options for pseudoaneurysms (PAs) of the head and neck. We explore different clinical situations in which these were selected for PA management at a single institution. *Methods*. Over a period of ten years, 33 patients presented to our hospital with PAs of the head and neck. Their outcomes and procedural complications are discussed. *Results*. We observed a complication rate of 18.2% (6 of 33), consisting predominantly of infarcts following vessel occlusion. As measured by the modified Rankin Scale, 25 (75.8%) patients had achieved favorable outcomes on discharge. A single patient who was treated with stent-assisted coiling expired following procedural complications. *Conclusions*. In our series, most patients with traumatic/iatrogenic PAs were successfully treated with parent vessel sacrifice. When parent vessel occlusion is not an option, stenting with or without coiling, or flow diversion, may also be safe and effective alternatives.

## 1. Introduction

The management of pseudoaneurysms (PAs) of the head and neck is challenging. A pseudoaneurysm can result from injury to a vessel that causes a disruption of the vessel wall. The resulting hematoma is encapsulated by the surrounding tissue [1]. At present, treatment options range from conservative treatment to microsurgery and endovascular therapy. While the current literature favors endovascular treatment of PAs over traditional surgical techniques, no treatment has proven infallible. Stenting is a commonly reported treatment, especially when the etiology is traumatic [2–4]. Selective coiling of the aneurysm with or without assistance and parent vessel sacrifice are also commonly used strategies. The purpose of the current study is to examine the different strategies for management of patients diagnosed with PAs.

## 2. Materials and Methods

Following Institutional Review Board approval, we reviewed the charts of 33 patients treated at our institution for pseudoaneurysms between 2003 and 2013. The mean age of the patients was 49, ranging from 19 to 82 years. The group consisted of 14 females (42.4%) and 19 males (57.6%). The causes of PA (Table 1) were traumatic in six cases (18.2%), iatrogenic in ten (30.3%), and idiopathic in seventeen (51.5%), with seven of the latter group presenting as subarachnoid hemorrhages (SAH) (21.2%). Mycotic aneurysms were excluded from the review. Clinical outcomes were determined using the modified Rankin Scale [5].

Parent vessel deconstruction was undertaken in 15 cases; eight of these were achieved by a combination of coils and Onyx embolization. PAs were iatrogenic in 6 of these cases and traumatic in 3. Five of the 7 patients presenting with SAH were treated with this method. In 13 of 15 patients treated with parent vessel deconstruction, the intervention was emergent. Eight of the 15 occurred in the ICA, six in the VA, and one in the lingual artery. When Onyx was used for embolization, the parent vessel was initially filled with a scaffolding of coils, onto which the embolic material was injected. This method spares the need for extensive coil deployment in order to occlude the vessel [6]. In all patients, collateral circulation was confirmed prior to embolization.

Selective coiling of the PA sac was undertaken in six patients. Two of the six PAs were in the ICA, one in the VA, one in the internal maxillary artery, one in the basilar artery, and one in the superficial temporal artery.

Six patients, all with PAs of the ICA, underwent stent-assisted coiling. Six patients were stented alone; just one of these was emergent. Two patients had PAs of the VA, and the other 4 had PAs of the ICA. Indications for stenting include having a fusiform morphology of the PA, or being wide-necked. The stent is deployed over the neck of the PA and may be self-expanding or balloon-expandable. When using a stent-assisted coiling technique, deploying the stent prior to coiling is preferable [7].

Several types of coils were used. Parent vessel deconstruction was performed with coils alone or with a combination of Onyx and coils. A variety of stents were used, namely, Pipeline (ev3, Irvine, California), Enterprise (DePuy, Warsaw, Indiana), Wingspan (Stryker, Fremont, California), Acculink (Abbott Vascular, Abbott Park, Illinois), and SMART (Cordis Endovascular, Bridgewater, New Jersey). In each case, the choice of stent was dependent on surgeon preference.

## 3. Results

The overall complication rate was 18.2% (6 of 33 patients). Included in this count is one patient who expired following intraoperative complications. The nature of the complications included infarcts and extravasation (Table 2). Regarding clinical outcome on discharge, patients were determined to have achieved favorable outcomes (mRS = 0–3) in 75.8% of cases (25 of 33).

Five patients whose vessels were sacrificed had procedural complications (33.3%). In each case, the complication consisted of one or more infarcts and resulted in poor outcomes for four patients (mRS = 4, 5). In one patient who had presented with a SAH and was treated with vessel sacrifice, there was a new hemorrhage recognized the day after the intervention, following which the patient expired. As the patient's ICA had been deconstructed and the new hemorrhage was noted along the tract of the ventriculostomy catheter, this was deemed unrelated to the patient's coiling procedure.

Of the six patients whose PAs were selectively coiled, one had to undergo vessel occlusion following failure of the initial procedure. This patient was discharged with an mRS of 4, indicating moderate to severe impairment requiring assistance with activities of daily living [5]. The remaining five patients had good outcomes.

Within the group of patients treated with stent-assisted coiling (*n* = 6), one died following extravasation during the procedure. The remaining 5 PAs were 100% occluded on follow-up. The patients who were only stented (*n* = 6) also had good outcomes (mRS = 0–3), with no complications.

## 4. Discussion

Most PAs were treated by endovascular means at our institution, with an acceptable complication rate and favorable outcomes. Although one patient expired from complications, this person presented with SAH, which notoriously carries a high rate of mortality [8].

Selective coiling is often not feasible or fails due to the PA morphology, such as when the lesion is wide-necked, in which case the parent vessel is sacrificed [9]. Failed selective occlusion occurred in one of our patients. Full vessel deconstruction may also be necessary if the reason for treatment is emergent, like an intraoperative rupture. One published case study describes the occurrence of profuse epistaxis caused by a ruptured PA of the ICA, following endoscopic sphenoid surgery. The patient refused vessel occlusion for fear of an ischemic attack, and the PA was selectively coiled. Subsequently, the patient experienced a nasal hemorrhage and expired as a result [10]. In our series, all four patients with PAs and active bleeding were successfully treated with parent vessel deconstruction (as mentioned above, selective coiling was attempted and failed in one of these four patients).

Vessel occlusion is a reliable strategy to secure PAs, but, as previously mentioned, it may lead to ischemia if there is no sufficient collateral flow [11]. In the present study, the majority of the complications (5 of 6) were due to infarcts following deconstructive coiling, though the presence of collateral circulation had been established, and this resulted in functional impairment in four cases (mRS = 4, 5). For patients who are experiencing a bleed from their PA, the benefit of vessel occlusion, especially using a combination approach with Onyx embolization, outweighs the risk of ischemia or thromboembolism. This technique allows for a rapid and thorough occlusion of the bleeding vessel [6].

Though they may not be actively bleeding, patients who present with an SAH and PA tend to undergo emergent vessel occlusion, due to the high risk of rebleed [12]. In one study of SAH treatment in 29 patients with vertebral or basilar dissection, 9 patients experienced a rebleed before endovascular management of their lesions could be attempted. Eight of these patients had pseudoaneurysms. Following treatment, there were no further hemorrhages in any patient [13]. Of our seven patients who presented with SAH and PA, occlusion of the vessel was chosen in five, and infarcts were only documented in one of these individuals (another one expired following rehemorrhage unrelated to surgical intervention). Two of seven SAH patients underwent vessel-sparing embolization of the PA, one of whom died following extravasation during the stent-assisted coil due to the fragile nature of the wall of those lesions.

The use of stents, either bare, graft, or in combination with coiling, as well as flow diversion devices, is attractive as they allow salvage of the parent vessel. However, these procedures carry the risk of thromboembolism and in-stent stenosis at follow-up [4, 14]. Cothren et al. found that patients who underwent stenting and anticoagulation therapy had a 45% rate of stenosis of the vessel on follow-up, compared with 5% of the conservative treatment group [3]. These results are at odds, though, with the findings of Berne et al. who found stenosis in only one of seven stented patients (vertebral and carotid arteries) who returned for follow-up [2]. As discussed, in our study, the one patient who had SAH and received stent-assisted coiling expired, but all other stented patients had good outcomes (mRS = 0–3). No further complications were reported in this group. One patient in this series was treated with a pipeline flow diversion device. This method has promise, though it requires dual antiplatelet therapy and exhibits delayed occlusion time [7, 15].

In previously published reports, stenting is the most frequently chosen treatment for traumatic PAs [2–4]. This was, however, not the case in the current study as only 2 of 6 courses of treatment for traumatic PAs involved stents. For traumatic/iatrogenic PAs with active bleeding or at high risk of rebleeding we prefer parent vessel sacrifice because this allows immediate and complete occlusion of the PA.

## 5. Conclusion

This study serves to report the course of treatment of 33 patients treated over ten years at a single institution. In our series, most patients with traumatic/iatrogenic PAs were successfully treated with parent vessel sacrifice. When parent vessel occlusion is not an option, stenting with or without coiling or flow diversion may also be safe and effective alternatives.

## Figures and Tables

**Table 1:** Pseudoaneurysm intervention: breakdown of location and etiology.

Management	Cases	Location	Etiology
Vessel deconstruction by coil and Onyx	8	ICA (4), VA (3), lingual	Iatrogenic (5), idiopathic (2), traumatic
Vessel deconstruction by coil	7	ICA (4), VA (3)	Idiopathic (4), traumatic (2), iatrogenic
Selective coiling	6	ICA (2), VA, Max, BA, STA	Idiopathic (3), iatrogenic (2), traumatic
Stent-assisted coiling	6	ICA (6)	Idiopathic (5), traumatic
Stent	6	ICA (4), VA (2)	Idiopathic (3), iatrogenic (2), traumatic

**Table 2:** Complications and revisions of pseudoaneurysm intervention.

Management	Emergent or elective	Complication or revision	Cases	Location	mRS
Vessel deconstruction by coil and Onyx	Emergent (1 SAH)	Infarct	3	ICA	4 (2), 5
Vessel deconstruction by coil	Elective	Infarct	2	ICA	0-1, 4
Selective coiling	Emergent	Revision-deconstruction by coil and Onyx	1	ICA	4
Stent-assisted coiling	Emergent (SAH)	Extravasation	1	ICA	6
Stent		N/A	0		
